# The Effects of Eugenol, Trans-Cinnamaldehyde, Citronellol, and Terpineol on *Escherichia coli* Biofilm Control as Assessed by Culture-Dependent and -Independent Methods

**DOI:** 10.3390/molecules25112641

**Published:** 2020-06-06

**Authors:** Magdalena A. Olszewska, Astrid Gędas, Manuel Simões

**Affiliations:** 1 Department of Industrial and Food Microbiology, Faculty of Food Science, University of Warmia and Mazury in Olsztyn, Plac Cieszyński 1, 10-726 Olsztyn, Poland; gedasastrid@gmail.com; 2 LEPABE–Department of Chemical Engineering, Faculty of Engineering, University of Porto, 4200-465 Porto, Portugal

**Keywords:** biofilm, confocal laser scanning microscopy, *Escherichia coli*, essential oil compounds, flow cytometry

## Abstract

Bacterial biofilms contribute to problems with preserving food hygiene, jeopardizing any conventional intervention method used by the food industry. Hence, the approach of using essential oil (EO) compounds effective in biofilm control has considerable merit and deserves in-depth research. In this study, the effect of selected EO compounds (eugenol, trans-cinnamaldehyde, citronellol, and terpineol) was assessed on *Escherichia coli* biofilm control by plate count, resazurin assay, and Syto^®^ 9/PI (-/propidium iodide) staining coupled with flow cytometry (FCM) and confocal laser scanning microscopy (CLSM). The selected EO compounds effectively inhibited the growth of planktonic *E. coli* at low concentrations of 3–5 mM, revealing a high antimicrobial activity. EO compounds markedly interfered with biofilms too, with trans-cinnamaldehyde causing the most prominent effects. Its antibiofilm activity was manifested by a high reduction of cell metabolic activity (>60%) and almost complete reduction in biofilm cell culturability. In addition, almost 90% of the total cells had perturbed cell membranes. Trans-cinnamaldehyde further impacted the cell morphology resulting in the filamentation and, thus, in the creation of a mesh network of cells. Citronellol scored the second in terms of the severity of the observed effects. However, most of all, it strongly prevented native microcolony formation. Eugenol and terpineol also affected the formation of a typical biofilm structure; however, small cell aggregates were still repeatedly found. Overall, eugenol caused the mildest impairment of cell membranes where 50% of the total cells showed the Syto^®^ 9+/PI– pattern coupled with healthy cells and another 48% with injured cells (the Syto^®^ 9+/PI+). For terpineol, despite a similar percentage of healthy cells, another 45% was shared between moderately (Syto^®^ 9+PI+) and heavily (Syto^®^ 9–PI+) damaged cells. The results highlight the importance of a multi-method approach for an accurate assessment of EO compounds’ action against biofilms and may help develop better strategies for their effective use in the food industry.

## 1. Introduction

Bacterial biofilm formation is a serious and ongoing concern in the food industry, mostly due to the ability of biofilm inhabitants to withstand adverse conditions [1,2]. The biofilm formation process is divided into separate stages and includes an attachment step of single cells to a surface, followed by microcolony formation and subsequent growth into mature biofilms with self-production of extracellular polymeric substances (EPS) [3]. They can be formed on all types of abiotic surfaces and also on foods [4]. Bacterial adhesion on surfaces and subsequent biofilm formation usually leads to the enhanced antimicrobial tolerance of biofilm cells, while the risk of food contamination and/or recontamination is increased [5]. Moreover, while biofilm is formed, cells can disperse and colonize other niches, spreading spoilage- and disease-causing microorganisms. Even though the food safety risks related to biofilms have been extensively studied in the past two decades [6,7,8,9,10,11,12], biofilm formation in the food industry is still poorly controlled.

Previous studies have shown how various food spoilage and pathogenic bacteria can persist due to biofilm formation either on food or in a food production environment, e.g., *Escherichia coli* O157:H7 [13], *Salmonella enteritidis* [5], *Listeria monocytogenes* [14], *Pseudomonas aeruginosa* [15], *Pseudomonas fragi* [16], *Bacillus cereus* [17], and *Lactobacillus plantarum* [18,19]. It is thus very difficult to control these bacteria within biofilms, despite applying the best antimicrobial agents [20]. Since neither of the methods guarantees total control, the search for new antimicrobials to be used along the food production chain has not yet been completed. A recent trend towards plant-based antimicrobials has become apparent as a result of consumer and authorities’ demands for natural alternatives to be used in the agriculture and food sectors [21,22,23]. Consequently, essential oils (EOs) and their compounds have gained great interest due to their natural origin as well as antimicrobial and antioxidant activities [4]. For instance, EOs such as linalool, carvone, and eugenol, have already been characterized as highly effective against a variety of Gram-positive and Gram-negative bacteria [24]. Moreover, the antimicrobial potential of carvacrol has recently been studied against *Escherichia coli* via a multi-faceted approach including the determination of membrane disruption, depolarization, and generation of reactive oxygen species [25]. Indeed, EOs may be applied directly to the food or incorporated into food packaging and intended to extend the food shelf-life [26]. On the other hand, the relatively low environmental toxicity of EOs makes them attractive alternatives to chemical disinfectants, contributing to a minimized risk of disinfectant resistance and even disinfectant and antibiotic cross-resistance due to their widespread and long-term use [27]. Thus, nowadays, there is a particular interest in understanding how these new antimicrobials can control microbial growth, particularly biofilms.

In this study, the antibiofilm potential of selected EO components was assessed on *Escherichia coli*. Cells of fecal indicatory bacteria, such as *E. coli*, contaminate food or water and survive in many environmental conditions due to different mechanisms, including adhesion to surfaces, thereby reducing the usability of conventional processing and chemical sanitizing methods in the food industry [28,29]. Eugenol, trans-cinnamaldehyde, citronellol, and terpineol were tested for their antimicrobial activity against planktonic cells and biofilms. Their antibiofilm effects were determined in terms of metabolic activity, culturability, and cell membrane integrity in combination with flow cytometry (FCM). Further elucidation of the antibiofilm action of the EO components was gathered by confocal laser scanning microscopy (CLSM) inspections of the biofilm structure.

## 2. Results

### 2.1. Antimicrobial Activity

The antibacterial activity of EO compounds was determined in terms of MIC (Table 1). The compounds that demonstrated lower MIC (3 mM) were eugenol and trans-cinnamaldehyde. Citronellol and terpineol had a MIC of 5 mM. Control experiments revealed no inhibitory effects (data not shown). In fact, other authors found DMSO could be bacteriostatic at higher concentrations (12.5%–20%, *v v*^−1^) [30,31].

### 2.2. Antibiofilm Tests–Resazurin Assay and Plate Counts

**Figure 1:**
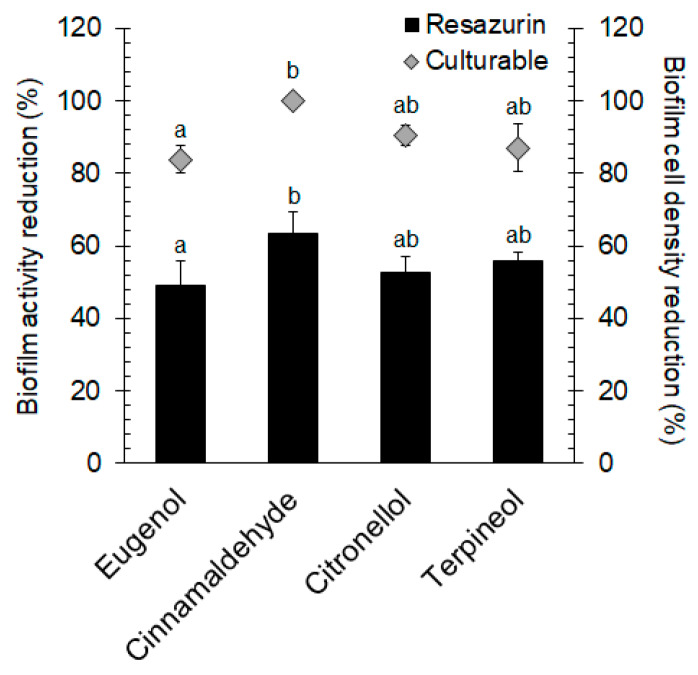
Effects of eugenol, trans-cinnamaldehyde, citronellol, and terpineol on biofilm cell metabolic activity and culturability of *E. coli* CECT 434. Different lowercase letters indicate a significant difference among essential oil compounds (*p* < 0.05).

Trans-cinnamaldehyde was the most effective EO compound at the concentration of 3 mM, causing more than 60% reduction in biofilm metabolic activity and almost complete reduction in biofilm cell culturability (Figure 1). However, significant reductions in biofilm cell metabolic activity and culturability were also caused by eugenol (3 mM; 49% and 84%, respectively), citronellol (5 mM; 53% and 90%, respectively), and terpineol (5 mM; 56% and 87%, respectively) (*p* < 0.05). Finally, we found a statistically significant difference between trans-cinnamaldehyde and eugenol in biofilm inactivation (*p* < 0.05) and culturability reduction (*p* < 0.05).

### 2.3. Antibiofilm Formation–FCM Supplemented by EFM

**Figure 2:**
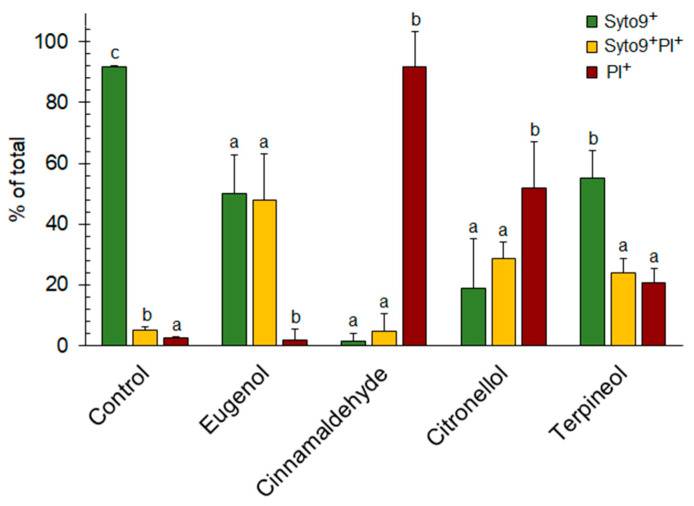
Percent of *E. coli* CECT 434 cells with healthy (Syto^®^ 9+PI–), moderately (Syto^®^ 9+PI+) and heavily (Syto^®^ 9–PI+) damaged membranes in antibiofilm formation experiments with eugenol, trans-cinnamaldehyde, citronellol, and terpineol. Different lowercase letters indicate a significant difference among essential oil compounds (*p* < 0.05).

FCM results provided information on the membrane integrity of biofilm cells which have been exposed to EO compounds. Figure 2 presents the percentage of cells differentiated into three levels of potential membrane damage.

Without EO compounds, 90% of the cells maintained cell membrane integrity (Syto^®^ 9+PI–) while 10% of the cells had injured (Syto^®^ 9+PI+) or damaged (Syto^®^ 9–PI+) cell membranes (*p* < 0.05). Bacteria exposure to EO compounds resulted in distinctive fluorescence patterns, as observed with flow cytometry. Cells with healthy membranes (Syto^®^ 9+PI–) were reduced by eugenol (3 mM) and terpineol (5 mM) to 50% and by citronellol (3 mM) and trans-cinnamaldehyde (5 mM) to 20% and 2%, respectively. In return, the percentage of injured (Syto^®^ 9+PI+) and/or damaged (Syto^®^ 9–PI+) cells increased. Following eugenol exposure, 48% of the cells were injured and did not differ significantly from Syto^®^ 9+PI– cells (*p* > 0.05), while almost 90% of the cells had damaged cell membranes from trans-cinnamaldehyde exposure, which also differed significantly from Syto^®^ 9+PI– cells (*p* < 0.05). In the case of citronellol and terpineol, 30%–50% and 25%–20% of the cells had moderately (Syto^®^ 9+PI+)–heavily (Syto^®^ 9–PI+) damaged cell membranes, and they differed significantly from Syto^®^ 9+PI–cells (*p* < 0.05) (Figure 2).

**Figure 3:**
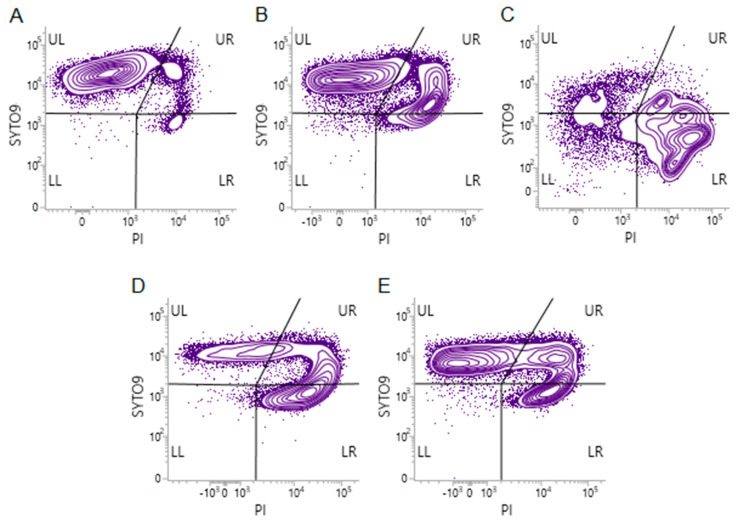
Flow cytometric analysis of *E. coli* CECT 434 treated with different EO compounds. Bacterial cell samples were stained with a mixture of Syto^®^ 9 and PI. The Figure shows different staining patterns of (**A**) untreated; (**B**) eugenol-treated; (**C**) trans-cinnamaldehyde-treated; (**D**) citronellol-treated; and (**E**) terpineol-treated cell samples. UL (upper-left), Syto^®^ 9+PI– and healthy cells; UR (upper-right), Syto^®^ 9+PI+ and moderately damaged cells; LR (lower-right), Syto^®^ 9–PI+ and severely damaged cells; LL, (lower-left), Syto^®^ 9–PI– and unknown cells.

**Figure 4:**
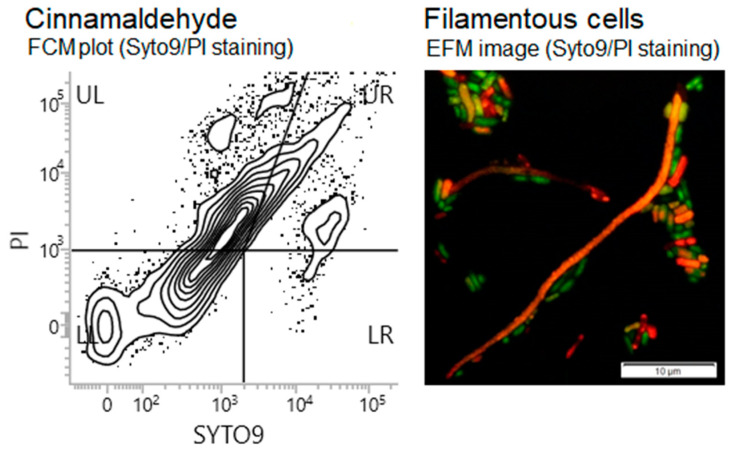
Flow cytometry (left) and epifluorescence microscopy (right) graphs of *E. coli* CECT 434 presenting a cell population with morphological variability including filamentous cells as affected by trans-cinnamaldehyde.

For eugenol, citronellol, and terpineol, a distinctive curve-shaped pattern was observed with emerging subpopulations of Syto^®^ 9+PI+ and Syto^®^ 9–PI+ cells. In the case of trans-cinnamaldehyde, we found a different fluorescence pattern, which raised a question on the extent to which this EO component is interfering (Figure 3). This was resolved by analyzing samples consisting of higher numbers of cells, with no cell size and particle size restriction, where the cell population has stretched out, suggesting the shape of the cells varied considerably (Figure 4). As a result, the EFM allowed visualizing long filamentous cells frequently stained red. However, ambiguous colors, like yellow or orange, were also observed that emphasize how challenging it is for these cells to maintain membrane integrity (Figure 4).

### 2.4. Antibiofilm Formation–CLSM

**Figure 5:**
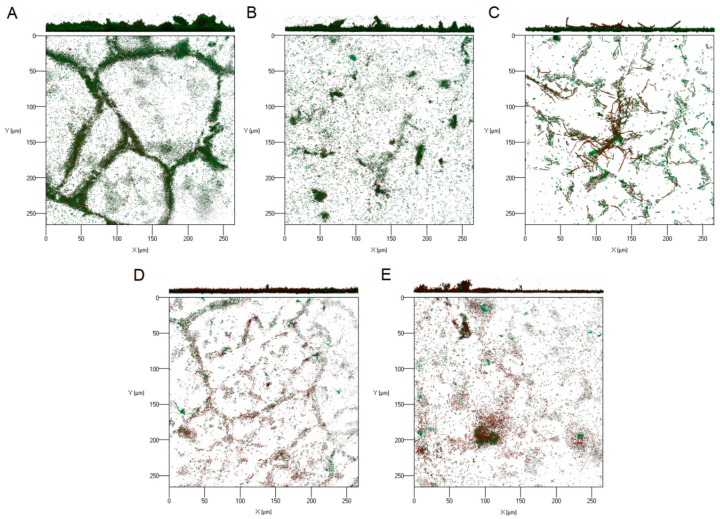
Confocal laser scanning micrographs of *E. coli* CECT 434 obtained from confocal z-stacks using ZEN 2.3 software. These images present an aerial view of biofilm structures, with the vertical projection on the above. The Figure presents (**A**) untreated; (**B**) eugenol-treated; (**C**) trans-cinnamaldehyde-treated; (**D**) citronellol-treated; (**E**) terpineol-treated sessile cells.

Representative biofilm structures observed using CLSM are shown in Figure 5. The images correspond to three-dimensional (3D) reconstructions obtained from confocal stacks, with the shadow projection on the above. Substantial variability in 3D biofilm architecture was observed following the exposure to the selected EO compounds. Without EO compounds, *E. coli* formed rough biofilms containing several small aggregates and of variable thickness resembling a mushroom-shaped structure (Figure 5A). Biofilms with eugenol (3 mM) were characterized by a relatively thin structure and small scattered cell clusters (Figure 5B). In turn, biofilms with trans-cinnamaldehyde (3 mM) repeatedly included cells of different sizes, with greatly elongated cells often fluorescing red and tying together and/or protruding from the biolayer (Figure 5C). No mushroom-like but rather a network of thin cell chains was observed in the biofilms with citronellol (5 mM), where red cells dominated over green cells (Figure 5D). The biofilms with terpineol (5 mM) revealed several cell aggregates with green cells located deeply inside and numerous red cells spread over the biofilm (Figure 5E).

**Figure 6:**
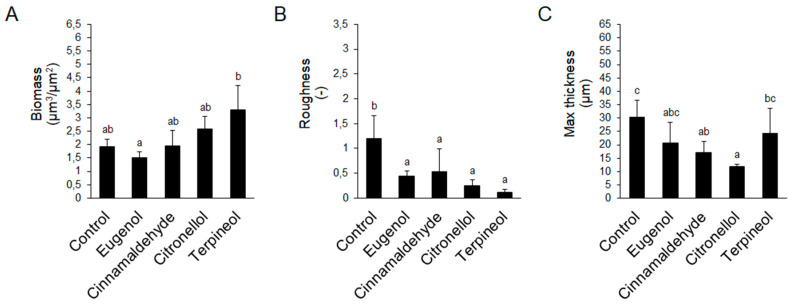
Biofilm structural parameters: the biomass (**A**), roughness (**B**), and maximum thickness (**C**) in antibiofilm formation experiments with eugenol, trans-cinnamaldehyde, citronellol, and terpineol on *E. coli* CECT 434. Different lowercase letters indicate a significant difference among essential oil compounds (*p* < 0.05).

Subsequently, the biomass, roughness, and maximum thickness parameters were extracted from confocal stack images to quantify biofilm structures (Figure 6). As for the untreated *E. coli*, its biofilm was characterized by relatively low biovolume (Figure 6A) and high roughness (Figure 6B), with an average maximum thickness of 30 µm (Figure 6C). The opposite trend was observed for the biofilms following exposure to EO compounds, with their roughness being significantly lower as compared to the untreated biofilms (*p* < 0.05) (Figure 6B). We also found a decline in maximum thickness, revealing the lowest values when they suffered from trans-cinnamaldehyde and citronellol exposures (17 and 12 µm, respectively) (Figure 6C).

## 3. Discussion

Since bacterial cells show remarkable complexity in the spatial organization of biofilms that determines their mode of persistence, the control of biofilm formation remains difficult [15]. Hence, the development of effective strategies presents a challenge for the food industry. There has been a great deal of research to better understand biofilm formation and to identify improved control strategies. Various products from the secondary metabolism of plants (phytochemicals) are of potential interest for antimicrobial applications due to their green status. EOs can exert an antimicrobial activity at low concentrations and as such they should not induce resistance mechanisms in bacterial cells [32]. The antimicrobial mechanisms of EOs are still not fully understood, but the currently accepted mechanisms include the cell membrane interaction and permeabilization, and the cytoplasmic pH decrease [21]. In this study, selected EO compounds effectively inhibited the growth of planktonic *E. coli* at low concentrations of 3–5 mM, revealing a high antimicrobial activity and thus potential usability in the food industry. For this reason, we used these EO concentrations in biofilm control experiments. It should be noted, however, that these compounds tend to show higher bactericidal concentrations, which did not exceed 10 mM. Considering effective EOs, the number of studies focusing on their effects on biofilm control has increased. A prior report has demonstrated the usefulness of thyme oil, which slowed down biofilm formation by *Staphylococcus aureus* and enhanced the efficiency of benzalkonium chloride (BAC) [33]. In another study, carvacrol and thymol interfered with adhesion and inhibited biofilm formation by *Pseudomonas aeruginosa* [34]. More recently, thymol and oregano revealed to be more effective in inhibiting biofilm formation by *E. coli* than carvacrol [35]. However, carvacrol and thymol showed higher activity against *Salmonella* biofilms than oregano did. Screening for antibiofilm agents among new EO compounds like sabinene, carveol, citronellol, and citronellal demonstrated variability between them in preventing *E. coli* biofilm setup [27]. However, these studies might have implications for food preservation, as EOs could be applied directly on the food surface or incorporated into food packaging wherein a migration of the compound occurs and controls the microbial action [26]. Indeed, the application of EOs in food packaging as natural inhibitors has received increasing attention in large part due to the consumers’ environmental awareness. The same applies to the chemical sanitizer substitutes that can reduce the incidence of foodborne spoilage and illnesses [21]. However, due to the multi-faceted nature of biofilm development and strong antimicrobial tolerance, it is important to better understand the effects of EOs on the biofilm traits. Therefore, we have applied a multi-method approach. Four EO compounds (eugenol, trans-cinnamaldehyde, citronellol, and terpineol) were selected to assess their effects on *E. coli* biofilms through culture-dependent and independent methods. In particular, plate counting was used to determine the reduction in culturable cell fraction of biofilms following exposure to EOs. As for culture-independent methods, a resazurin assay was employed to assess the reduction in biofilm metabolic activity and Syto^®^ 9/PI staining was used to determine the membrane damage of cells that following dispersal were precisely analyzed at the single-cell level on a flow cytometer (FCM). Finally, CLSM was used to compare the effects of EO compounds on the disruption/decomposition of the spatial organization of biofilms, which allowed to deepen the understanding of biofilm structure–function relationships.

EO compounds markedly interfered with biofilms, with trans-cinnamaldehyde causing the most prominent effects. The antibiofilm activity of trans-cinnamaldehyde was demonstrated by a high reduction of cell metabolic activity and culturability, as well as a loss of membrane integrity by biofilm cells, which tended to show different morphology, i.e., filamentous cells and weakened coverage of the substratum. Eugenol revealed different antibiofilm activity, causing the lowest biofilm cell metabolic activity and culturability reductions. Besides, FCM showed that half of the cells experienced injury whereas the other half retained their membrane integrity as well as the partial microcolony organization, as visualized by CLSM. Importantly, cinnamaldehyde and eugenol are the two most predominant compounds of cinnamon oil, and both are considered as promising natural and safe (GRAS status) ingredients for the use in the food industry due to their effectiveness against foodborne pathogens and spoilage bacteria and stability under food processing conditions [36,37,38]. Cinnamaldehyde was reported as an effective antibacterial active compound in edible films used, for example, in the baked wrapped chicken [39]. Following formulation into polymeric films, eugenol was able to impair biofilm formation by *E. coli* and seemed crucial in reducing bacterial biofilms according to the literature [40]. In our study, cinnamaldehyde had a superior effect over eugenol and was also considered as an important inducer of cell elongation, suggesting its interference with the cell replication mechanism. What happens is that stressed cells continue to grow but stop dividing. Once the stress is over, the filaments start dividing again, producing smaller cells [41]. Here, many filamentous cells were characterized by substantial elongation and cell membrane perturbations. CLSM suggested these filamentous cells constitute a great part of the community allowing the construction of biofilms in which they are tied together and create a knit network at the expense of regular three-dimensional organization of cells. This also forces them to protrude from the biolayer, which may have a relevant function, such as the protection of smaller cells. Interestingly, filamentous *E. coli* cells were observed to deform upon hydrodynamic forces as investigated in a microfluidic device, regardless of whether they were growing or non-growing cells due to their plasticity [42]. In turn, it was shown that cells of *Vibrio cholerae* filamenting strain gained a competitive advantage in colonizing and spreading on particles of chitin, the material *Vibrio* species depend on for growth [43]. Filamentation allowed the creation of a mesh network of cells through physical entanglement and independent of cell–cell adhesion components and the major polysaccharide for the *V. cholerae* biofilm matrix. This cell network was also prone to fast surface spreading and subsequent dispersal. Indeed, the study provides a profound significance of how cell morphology affects bacterial success during surface colonization, competition within biofilms, and dispersal to new resource areas. The link between this biofilm architecture and the persistence of *E. coli* on surfaces at different residing sites remains open for future work. However, the locations where nutrients and moisture accumulate within different environmental settings, including food processing surfaces, are of greatest concern, because of the risk of filaments escaping and contaminating other surfaces and, subsequently, food.

Thus, two more EO compounds were taken under consideration, citronellol and terpineol, which were effective at the concentration of 5 mM against planktonic cells. They were able to cause a considerable loss of membrane integrity of biofilm cells; however, citronellol impacted them more severely. The target site for EOs seems to be the cell membrane [21]. Bacterial cells with damaged membranes often fail to attach and form biofilms [44]. Therefore, these monoterpene-based oils could be suitable biofilm control agents, especially citronellol as it diminished the formation of native biofilm structures predominated by the PI-positive cells. An important aspect related to both compounds is that they are approved by the FDA (Food and Drug Administration, USA) for food use. Before considering the use of EOs for food purposes, a balance needs to be carefully found between the dosage and the risk of toxicity [26]. However, they can be present in formulas of cosmetics, perfumes, and cleaners, whereas citronellol is also used on food crops as an active compound of pesticides [27]. When formulated into a disinfectant solution also lemongrass oil, which is mainly composed of cyclic and acyclic monoterpenes, showed powerful anti-biofilm effects, being a promising tool for reducing microbial colonization of food processing surfaces [45]. Due to their strong interference with biofilm cell membrane integrity, another valuable consideration may be given to investigations of the potential antimicrobial efficacy of these antimicrobials alone or in combinations with other treatments on vegetable produce [46]. Although further studies are needed to conclude on their suitability as natural food sanitizers, the antibiofilm activity presented by citronellol promises its use in sanitizing formulations. To go one step forward, the phenomenon of injured cells that frequently emerged here needs particular attention. These cells are not recoverable on selective media, which are typically employed in standard detection procedures. Thus, their detection should be considered to ensure sanitizer effectiveness.

## 4. Materials and Methods

### 4.1. Bacterium

*E. coli* CECT 434 was obtained from the Spanish Type Culture Collection (CECT). This strain has been used as a model microorganism for antimicrobial and antibiofilm testing [27,47,48]. The strain stored at −80 °C was subcultured onto Tryptic soy agar (TSA; Merck, Darmstadt, Germany) through incubation at 30 °C for 24 h, and before the experiments inoculated into TSB and grown overnight (14 h) at 30 °C and under agitation (150 rpm).

### 4.2. Phytochemicals

Trans-cinnamaldehyde, eugenol, and terpineol were obtained from Sigma-Aldrich (Lisbon, Portugal); citronellol was obtained from Acros Organics (Morris, NJ, USA) (Table 1). The EOs were dissolved in dimethyl sulfoxide (DMSO, Sigma-Aldrich, St. Louis, MO, USA). Control experiments were performed to ascertain the growth inhibitory effects in DMSO (5%–10%, *v v*^−1^).

### 4.3. Determination of Minimal Inhibitory Concentration

The MIC of EO compounds was determined by the microdilution method in a sterile 96-well microtiter plate [49]. An overnight cell culture was adjusted to a cell density of 10^6^ CFU mL^−1^ and transferred into a sterile 96-well polystyrene microtiter plate (Orange Scientific, Braine-l’Alleud, Belgium) with the different EO compounds at several concentrations (1, 3, 5, 8, 10, 13, 15, 18, and 20 mM) in a final volume of 200 μL. The antimicrobial solutions did not exceed 5% (*v*/*v*) of the well. Bacterial suspensions with DMSO and bacterial suspensions without EOs were used as negative controls. The plate was then incubated for 24 h at 30 °C and MIC corresponded to the concentration in which the final optical density (OD) was inferior or equal to the initial OD.

### 4.4. Biofilm Formation

Aliquots (200 μL) of a bacterial suspension in quarter-strength TSB, containing ~10^6^ CFU mL^−1^, were transferred into wells of a 96-well polystyrene microtiter plate and the plate was then incubated for 3 h at 30 °C under static conditions [27]. After that, the planktonic bacteria were removed and each well was then washed twice with NaCl solution (8.5 g/L) to remove the loosely attached cells. Subsequently, 190 µL of the TSB with 10 µL of either DMSO (control) or particular EOs at MIC was added and the plate was then incubated for 24 h at 30 °C under static conditions. The experiment included four replicate wells and was repeated twice using independent bacterial cultures.

### 4.5. Resazurin Assay

The resazurin microtiter plate assay was performed according to the literature [47]. Resazurin was obtained from Sigma-Aldrich (Portugal). This is a non-fluorescent blue dye that is reduced by the living cells to a red fluorescent resorufin and serves as an indicator of cell metabolic activity. Firstly, the wells were filled with 190 μL of sterile broth and 10 μL of resazurin solution (0.1 mg/mL). After 20 min of incubation at room temperature, in the dark, fluorescence (λ_ex_: 570 nm and λ_em_: 590 nm) was measured using a microtiter plate reader (FLUOstar Omega, BMG Labtech, Ortenberg, Germany). After measuring the fluorescence, the percentage of biofilm metabolic reduction was calculated as follows: [(FLUOcontrol – FLUOEOs)/FLUOcontrol] × 100.

### 4.6. Plate Counts

The assessment of biofilm cell culturability was performed according to the literature [27]. Firstly, the content of each well was harvested by scraping the surface carefully with a sterile pipette tip—the mechanically-detached biofilm bacteria—and transferred into 2-mL Eppendorf tubes followed by a vigorous (250 rpm) agitation for 1 min. Subsequently, biofilm cell suspensions (10 µL) or appropriate dilutions in NaCl (8.5 g/L) were drop-plated in duplicate onto TSA plates. The colonies on the plates were counted after 24 h of incubation at 30 °C and the percentage of biofilm cell density reduction was calculated as follows: [(CFU cm^−2^_control_ – CFU cm^−2^_EOs_)/ CFU cm^−2^_control_] × 100.

### 4.7. Biofilm Cell Membrane Integrity and FCM Supplemented by EFM

To study the membrane integrity status of biofilm cells, they were stained with two fluorescent dyes, Syto^®^ 9 and propidium iodide (PI) (LIVE/DEAD BacLight™ viability kit from Molecular Probes, LifeTechnologies, Eugene, OR, USA) as described in the literature [19]. For this purpose, the wells were rinsed with sterile NaCl (8.5 g L^−1^) and refilled with NaCl containing 3 µM Syto^®^ 9 and 20 µM PI. The plate was then incubated in the dark at room temperature for 15 min to enable the fluorescent labeling of the bacteria. The staining principle is based on the difference in cell membrane penetration by Syto^®^ 9 and PI. Syto^®^ 9 penetrates both live and dead cells due to its high permeability, whereas PI can only penetrate cells with impaired membranes due to its high molecular mass. Thus, live cells with intact membranes (Syto^®^ 9+PI–) fluoresce green, while dead cells with damaged membranes (Syto^®^ 9–PI+) fluoresce red. As intermediate states with different concentrations of both stains are often detected by flow cytometry, the fluorescence intensity of strong green and strong red cells (Syto^®^ 9+PI+) as well as weak green and weak red cells (Syto^®^ 9–PI–) could also be determined. Following harvesting and agitation, the well contents were analyzed with the use of a BD FACSLyric™ flow cytometer (Becton Dickinson, San Jose CA, USA) equipped with two lasers: blue (488-nm, air-cooled, 20-mW solid-state) and red (633-nm, 17-mW HeNe). The green fluorescence from the Syto^®^ 9-labeled cells was detected at the FL1 channel (530 ± 30 nm), whereas red fluorescence of the PI-labeled cells at the FL3 channel (630 ± 22 nm). BD FACSFlow™ solution (Becton Dickinson) was used as the sheath fluid and the BD™ CS&T Beads (Becton Dickinson) served for monitoring the consistency of the instrument optical alignment. All bacterial analyses were performed at the low rate settings, and the total event counts of 50,000 were acquired. The data were analyzed using dot plots, i.e., bivariate displays in which each dot represents one measured event by the BD FACSSuite V1.3 software (Becton Dickinson). For cell morphology examinations, the cells were visualized under an Olympus BX51 epifluorescence microscope (EFM) (Olympus, Hamburg, Germany) with the CellSens Dimension 1.5 software (Olympus, Hamburg, Germany) as described elsewhere [50].

### 4.8. Biofilm Formation in Lab-Tek™ and CLSM Analysis

As described above, the inoculum of *E. coli* was added to 4-well chamber slides (Nunc™ II; Lab-Tek™; Fisher Scientific, Waltham, MA, USA) at 800 µL per well, allowed to attach at 30 °C for 3 h, and then to form biofilms either with and without EOs at 30 °C for 24 h. After that, biofilms were stained with the LIVE/DEAD BacLight™ viability kit (Life Technologies) as described in the literature [51]. After staining, the chambers were detached from the slide and the NaCl solution (8.5 g L^−1^) was added to the remaining biofilms separated from each other with a gasket. A coverslip was then placed on the gasket, and BacLight™ mounting oil (Molecular Probes) was used to seal its corners, whereas nail polish was used to seal the slide. Images were acquired with a Zeiss LSM 800 confocal laser scanning microscope (Carl Zeiss Microscopy, Thornwood, NY, USA). All biofilms were scanned using a water-immersion objective lens (Zeiss, 40 × C Pan-Apochromat, NA 1.3) with a 488-nm argon laser and a 561-nm diode-pumped solid-state laser. The fluorescence was recorded within the range from 500 to 600 nm to collect green fluorescence and from 610 to 710 nm to collect red fluorescence. Four stacks of horizontal plane images (260 × 260 µm) with a z-step of 0.8 µm were acquired for each biofilm at different areas in the well. Serial images were captured and processed by Zeiss Zen 2.3 software (Carl Zeiss). Quantitative structural parameters (biovolume, roughness, and maximum thickness) were extracted from the confocal image series with COMSTAT 2, an image analysis software (www.comstat.dk) developed and described in the literature [52,53].

### 4.9. Statistics

All statistical analyses (analysis of variance—ANOVA) were performed using Statistica software ver. 13.1 (StatSoft Inc., Tulsa, OK, USA). Differences were considered significant at a significance level of *p* < 0.05.

## 5. Conclusions

In conclusion, the antibiofilm effects of EO compounds on *E. coli* were assessed with several approaches, including culture-independent methods, with trans-cinnamaldehyde and citronellol causing remarkable effects. This study provided a significant amount of data at the single cell and community levels, contributing to a better understanding of the effects of EO compounds against biofilms, and thus helping to make knowledge-based decisions for the use of EOs in the food industry. The employment of a multi-method approach is potentially relevant to understand the dynamics of bacterial biofilm formation and to develop reliable biofilm control strategies. Trans-cinnamaldehyde is highlighted for the relevant biofilm control action and for its use against biofilms through appropriate elimination and/or prevention of filament accumulation.

## Figures and Tables

**Table 1:** MIC values of selected essential oil (EO) compounds against planktonic *E. coli* CECT 434.

EO Compound	Class	MIC (mM)
Eugenol	Essential oil (phenol)	3
Trans-cinnamaldehyde	Essential oil (aldehyde)	3
Citronellol	Essential oil (alcohol)	5
Terpineol	Essential oil (alcohol)	5
